# Supplementary material for: The interactome of intact mitochondria by cross-linking mass spectrometry provides evidence for coexisting respiratory supercomplexes
Source: Mol Cell Proteomics. 2017 Dec 8;17(2):216–32. doi: 10.1074/mcp.RA117.000470 (PMC5795388; doi:10.1074/mcp.RA117.000470)
Supplement: Supplemental Data [file supp_RA117.000470_133922_0_supp_23978_fzffwf.zip › spectra_annotation/mito_spectra_annotation/381-1-11-1-14-1.pdf]

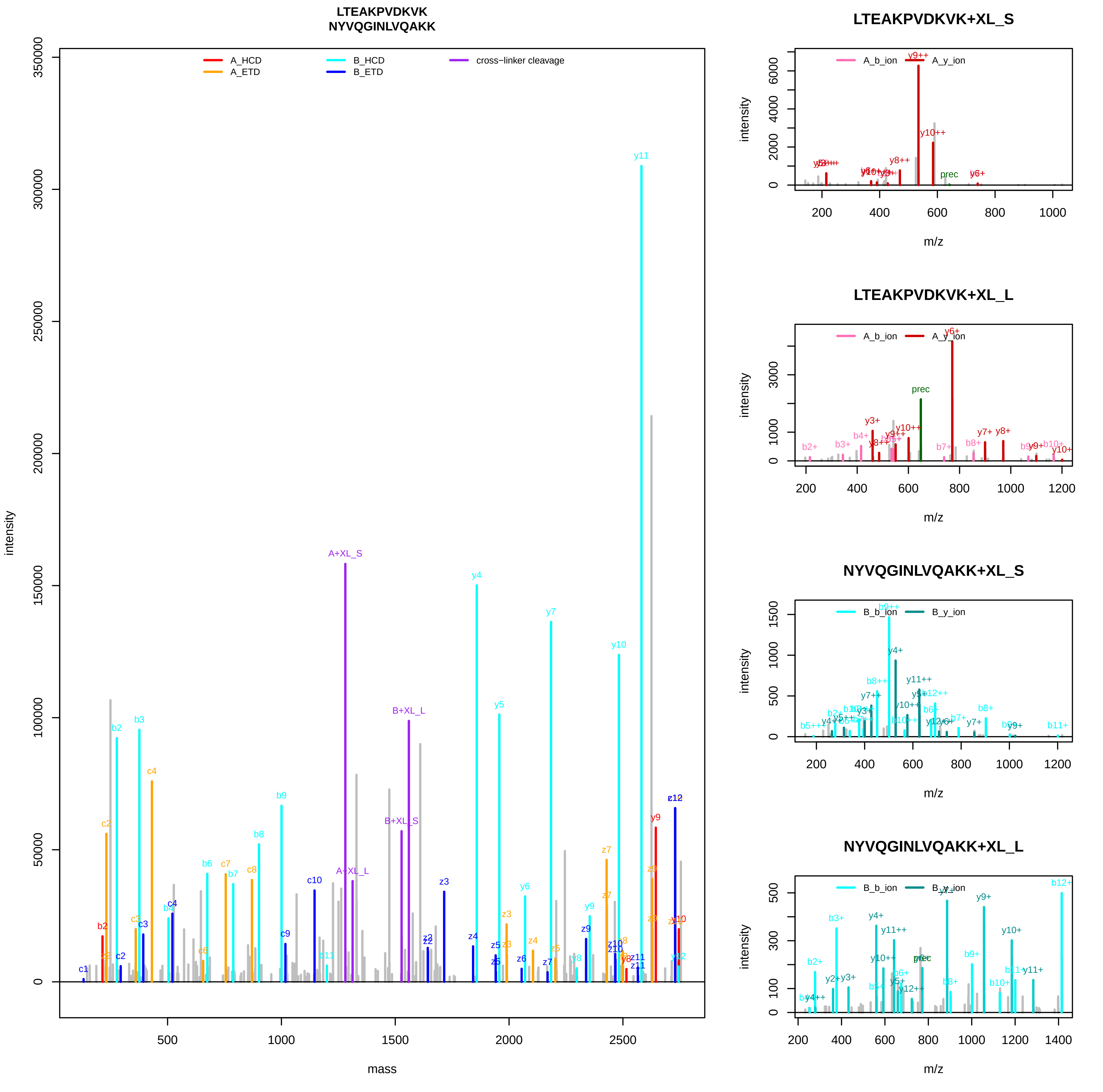
LTEAKPVDKVK
NYVQGINLVQAKK
A_HCD
A_ETD
B_HCD
B_ETD
cross−linker cleavage
intensity
mass
A+XL_S
A+XL_L
B+XL_L
B+XL_S
y11
y4
y7
y10
y5
y9
y6
b2
b3
b6
b7
b8
b9
b4
c4
c2
c3
c7
c8
c10
c9
c12
z3
z4
z5
z6
z7
z9
z10
z11
LTEAKPVDKVK+XL_S
LTEAKPVDKVK+XL_L
NYVQGINLVQAKK+XL_S
NYVQGINLVQAKK+XL_L
A_b_ion
A_y_ion
B_b_ion
B_y_ion
prec
m/z
